# Reappraisal of gap analysis for effusive crises at Piton de la Fournaise

**DOI:** 10.1186/s13617-021-00111-w

**Published:** 2022-01-10

**Authors:** Aline Peltier, Magdalena Oryaëlle Chevrel, Andrew J. L. Harris, Nicolas Villeneuve

**Affiliations:** 1 Université de Paris, Institut de Physique du Globe de Paris, CNRS, F-75005 Paris, France; 2 Observatoire Volcanologique du Piton de la Fournaise, Institut de Physique du Globe de Paris, F-97418 La Plaine des Cafres, France; 3 Laboratoire Magmas et Volcans, Université Clermont Auvergne, CNRS, IRD, OPGC, F-63000 Clermont-Ferrand, France; 4 Laboratoire Géosciences Réunion, Université de La Réunion, F-97744 Saint Denis, France

**Keywords:** Lava flow damage, Volcanic hazard, Basaltic eruption, Crises management, Volcano observatory

## Abstract

Effective and rapid effusive crisis response is necessary to mitigate the risks associated with lava flows that could threaten or inundate inhabited or visited areas. At Piton de la Fournaise (La Réunion, France), well-established protocols between Observatoire Volcanologique du Piton de la Fournaise – Institut de Physique du Globe de Paris (OVPF-IPGP) and civil protection, and between scientists of a multinational array of institutes, allow effective tracking of eruptive crises and hazard management embracing all stakeholders. To assess the outstanding needs for such responses Tsang and Lindsay (J Appl Volcanol 9:9, 2020) applied a gap analysis to assess research gaps in terms of preparedness, response and recovery at 11 effusive centers, including Piton de la Fournaise. For Piton de la Fournaise, their gap analysis implied widespread gaps in the state of knowledge. However, their work relied on incomplete and erroneous data and methods, resulting in a gap analysis that significantly underrepresented this state of knowledge. We thus here re-build a correct database for Piton de la Fournaise, properly define the scope of an appropriate gap analysis, and provide a robust gap analysis, finding that there are, actually, very few gaps for Piton de la Fournaise. This is a result of the existence of a great quantity of published work in the peer-reviewed literature, as well as frequent reports documenting event impact in the local press and observatory reports. At Piton de la Fournaise, this latter (observatory-based) resource is largely due to the efforts of OVPF-IPGP who have a wealth of experience having responded to 81 eruptions since its creation in 1979 through the end of September 2021.

Although welcome and necessary, especially if it is made by a group of scientists outside the local management of the volcanic risk (i.e., a neutral group), such gap analysis need to be sure to fully consider all available peer-reviewed literature, as well as newspaper reports, observatory releases and non-peer-reviewed eruption reports, so as to be complete and correct. Fundamentally, such an analysis needs to consider the information collected and produced by the volcano observatory charged with handling surveillance operations and reporting duties to civil protection for the volcano under analysis. As a very minimum, to ensure that a necessarily comprehensive and complete treatment of the scientific literature has been completed, we recommend that a third party expert, who is a recognized specialist in terms of research at the site considered, reviews and checks the material used for the gap analysis before final release of recommendations.

**Supplementary Information:**

The online version contains supplementary material available at 10.1186/s13617-021-00111-w.

## Introduction

Classically applied in business management, engineering, manufacturing and industry (e.g., Kotabe and Czinkota 1992; Chow and Hg 2007; The Art of Service 2021), gap analysis involves a comparison between actual and desired performance. Langford et al. (2007) add that “gap analysis is an assessment tool that compares a system’s actual performance with its potential”. It is an approach also applied in research settings from agriculture through medicine to geology (e.g., Bleiweiss 1998; van Ittersum and Cassman 2013; Begasse de Dhaem et al. 2020). The methodology and data used comes in a great variety of forms, but when applied to a research setting it involves comparing the actual state of knowledge and understanding regarding a phenomenon or process, and the desired or required level of knowledge. The upshot is the identification of fundamental gaps in our state of knowledge that need to be filled (cf. El Fadel et al. 2013), or gaps in data sets to be validated as real or artefacts (cf. Bleiweiss 1998). It is with this aim that Tsang and Lindsay (2020) applied a gap analysis to assess research gaps related to effusive basaltic eruptions in terms of preparedness, response and recovery.

The gap analysis of Tsang and Lindsay (2020) was carried out for 38 basaltic lava flow crises that threatened and/or inundated inhabited areas worldwide between 1950 and 2019, but suffered from incomplete data to inform their analysis. The analysis was based on:

“a literature review to collate formally and informally published data and accounts of basaltic lava flows that have threatened or impacted settlements or their supporting networks … (with the objective) … to extract lessons about how community understanding of volcanic hazards influences community resilience, (and) how lava flow modelling can inform planning”.

As part of this, key outputs were tabulations of all basaltic effusive events that threatened and/or inundated inhabited areas during 1950–2019 for 11 effusive centres, including Piton de la Fournaise (La Réunion, France), with each table listing impacts, responses and recovery actions for each of the 38 crises examined. Tsang and Lindsay (2020) then used the data tables to support a gap analysis, which involved a final table identifying “research gaps” in terms of effusive crisis documentation, monitoring, response, communication, evacuation and recovery at the 11 centres examined. The dataset used for the case of Piton de la Fournaise is unfortunately not well defined, incomplete and erroneous; a problem which results in an inaccurate gap analysis.

In this communication, we therefore first detail the well-established response protocol that is in place at Piton de la Fournaise to manage effusive crises and the potential for lava flow inundation, and use data published as part of this effort to correct the errors of Tsang and Lindsay (2020), the detail of which are given in the Electronic Supplementary Material. We then provide a complete database and descriptions of the eruptions that properly considers the great body of peer-reviewed literature that exists for effusive events at Piton de la Fournaise and that were overlooked by Tsang and Lindsay (2020). This database is capable of supporting a proper gap analysis and its construction demonstrates the rigor that must be applied to a literature search if a gap analysis is to be valid. In parallel, we more clearly define the steps involved in, and the scope of an, appropriate gap analysis for application of lava flow hazard assessment, community planning and mitigation at an effusive center. This includes clarification on the detail of effusive crisis response, evacuation and damage caused by effusive events at Piton de la Fournaise since 1950, plus lava flow model-based support of syn-crisis risk assessments that has been developed since 2014 (cf. Harris et al. 2019; Peltier et al. 2018, 2020). We use the database to re-implement the gap analysis, showing how an incomplete consideration of the literature can severely bias an analysis to the detriment of the host volcano observatory. We finish by proposing protocols for appropriate gap analysis application and scope when assessing research gaps in volcanic hazard, risk, impact and mitigation, and raise a serious concern regarding support for observatory operations if otherwise well-intended gap analyses are poorly set-up, executed and validated. Fundamentally, gap analyses need to follow well-defined and appropriate protocols, standards and criteria, and be utterly rigorous (Langford et al. 2007); these being two key issues that we address here.

## Piton de la Fournaise: effusive crisis response protocol and report dissemination

**Fig. 1:**
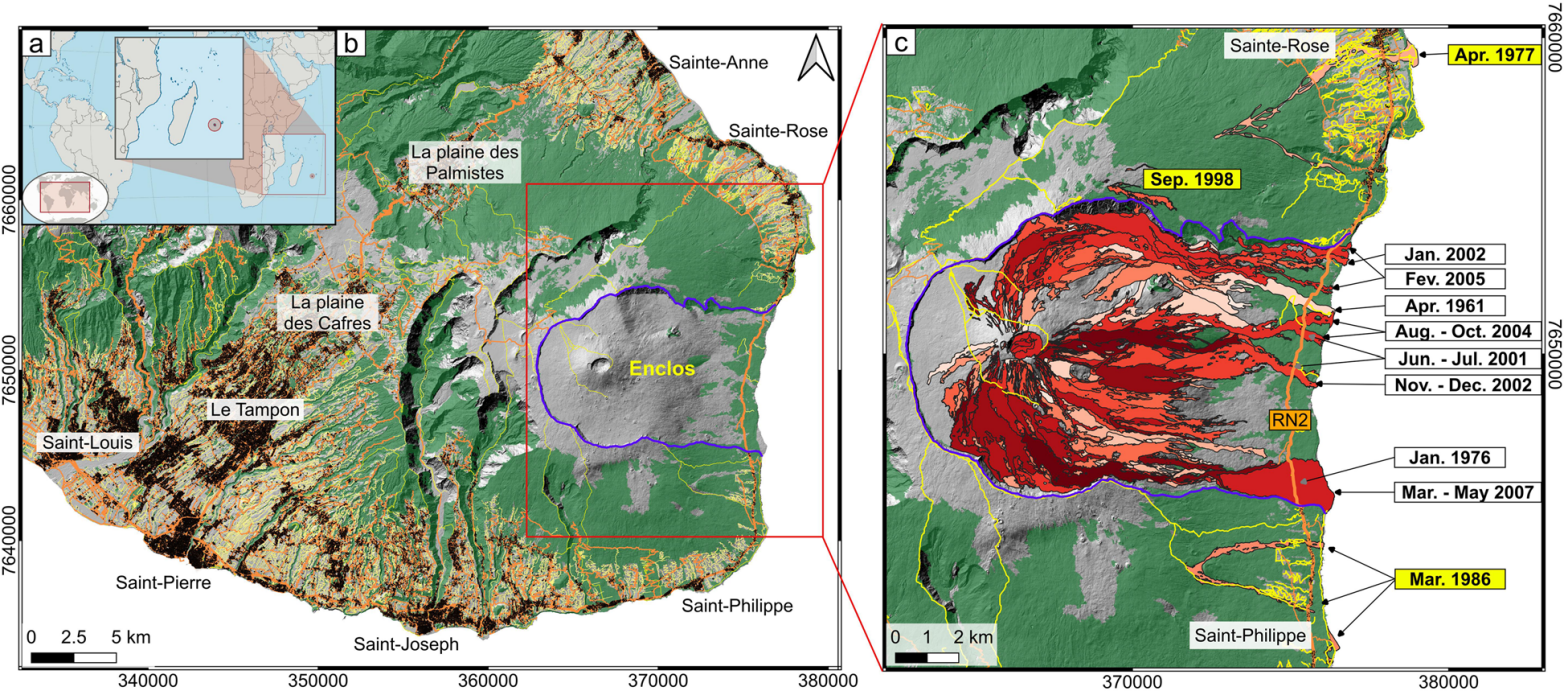
**a** Location of La Réunion island (credits Wikimedia Commons, the free media repository). **b** Map of Piton de la Fournaise showing the municipalities and towns (buildings are in black), roads (orange lines), trails (yellow lines), the vegetated areas (in green) and the location of the Enclos (blue line). The background is the hill shade of the lidar DEM from the Institut national de l’information géographique et forestière (IGN) – released in 2010, and coordinates are within system WGS84-UTM 40S. Buildings, roads, and trails are from BD TOPO® IGN. **c** Zoom of the Enclos and surrounding areas showing the lava flows from 1950 to 2021 (white to dark red). Lava flows that crossed the road are noted (grey arrow indicate flows that are now buried by more recent flows) and the Hors Enclos flows are highlighted with a yellow background

Piton de la Fournaise (La Réunion island, Indian Ocean, Fig. 1a) is a highly active dominantly effusive volcano, which has experienced two eruptions per year on average since the creation of Observatoire Volcanologique du Piton de la Fournaise – Institut de Physique du Globe de Paris (OVPF-IPGP) in 1979 (Peltier et al. 2009; Roult et al. 2012; Chevrel et al. 2021). Many actions are implemented by OVPF-IPGP to mitigate hazard prior to and during any eruption at Piton de la Fournaise, where operations carried out by the OVPF-IPGP are mandated by the French government via a national emergency plan. A government-mandated and legally-binding “plan of emergency actions” has been in existence since 1977, and it is operated before and during any and every eruption (e.g., Harris et al. 2017a; Peltier et al. 2018, 2020). This emergency plan (*Organisation de la Réponse de Sécurité Civile* (*ORSEC) - volcan du Piton de la Fournaise* emergency plan) states that OVPF-IPGP must inform the civil protection department of the *Préfecture* (i.e., the decentralized administrative service of the French government) of any changes in volcanic activity. OVPF-IPGP must also provide detail of that activity and communicate through regular, publically available, reporting. Since its creation in 1979 through September 2021, OVPF-IPGP had anticipated and responded to 81 eruptions, always up grading the alert subject to deformation and seismic trends typically several hours or days before eruption onset (Peltier et al. 2018). The imminence of an eruption is communicated to the Préfecture using a monitoring system based on a well-established and continuously-expanding instrument network (seismic, deformation, gas). This network has increased from five permanent, continuously monitoring stations as installed in 1981, to 101 by 2020 (Bachèlery et al. 1982; Peltier et al. 2009, 2018; Roult et al. 2012). The decision to change alert level, issuance of advisories, execution of safety measures and communication to all actors (such as town councils, local police, central authorities, and media) remains the sole responsibility of the *Préfet* (the head of the *Préfecture and representative of the French government in regions*).

When there is no alert level, hikers are free to go anywhere in the main caldera (an area of 100 km^2^, here after called “the Enclos”, Fig. 1) and even hike off of the marked trails. When a situation, determined to be “critical”, develops (rock falls, still hot (but cooling) lava flows, increase of seismicity or deformation) a pre-alert level (called *Vigilance*, i.e., “watch” in French) is issued. The intention of this alert level is to limit access to the volcano, with access being reduced to only authorized, marked trails. This alert level concentrates hikers to one site and well-defined trails, thus facilitating ease of location, evacuation and search and rescue. When an eruption becomes imminent (Alert 1), access to the Enclos and to the summit is closed and hikers present on the trails are evacuated. If an eruption starts inside the Enclos (Alert 2.1 or 2.2 depending on the location of the fissures), in theory all hikers should have already been evacuated. If, however, some remain in the danger zone (which can happen when dyke propagation to the surface is very short) then search and rescue is triggered. Until 2010, on occasion, the Enclos had been reopened during an eruption to allow the public to approach and view active lava flows. However, between 2014 and 2017 only journalists and photographers were allowed access during Alert level 2. Since 2017, access has been restricted further to include only emergency services and OVPF-IPGP staff. If an eruption starts outside of the Enclos (Alert 2–3), any population centres under threat are evacuated (see Fig. 1a for building and town locations). Since 2015, OVPF-IPGP has also been responsible for the provision of volcanic activity information relevant to civil aviation to the VAAC-Toulouse (Volcanic Ash Advisory Centres) by submitting a VONA (Volcano Observatory Notice for Aviation) during any change in activity.

Since 2014, a multi-national (involving OVPF-IPGP, Laboratoire Géosciences Réunion-IPGP, Université Clermont Auvergne – Observatoire de Physique du Globe de Clermont Ferrand, University of Turin, University of Pittsburgh, INGV Pisa) protocol has been implemented. This is based on rapid process of field, airborne and satellite-derived data to deliver near real-time assessments of lava flow propagation (Harris et al. 2017a, 2019; Coppola et al. 2020; Peltier et al. 2020). Through this protocol, all necessary source terms (i.e., time-averaged discharge rate and fissure location) required by the lava flow model initialized for Piton de la Fournaise (i.e., Chevrel et al. 2018) are collected to compute the most probable lava flow path and runout distances (Harris et al. 2017a, 2019). Mainly based on remote sensing data, this protocol is particularly effective if field access is limited or difficult, as demonstrated during the April 2020 eruption that occurred during the strictest lockdown related to COVID-19 (Peltier et al. 2020). The protocol allows communication of maps of the probable lava flow paths to the *Préfecture* to support response decisions, allowing the *Préfecture* also to assess the risk of fires in vegetated areas at lower elevations, as well as the likelihood of lava flow cutting the coastal road or entering populated zones (Harris et al. 2017a, 2019). In the event of an eruption outside the Enclos, towns and housing units in the flow path have been identified, as well as roads and other infrastructure, such as power lines (cf. Fig. 1). In addition since 2018, OVPF-IPGP uses the probabilistic maps to evacuate monitoring stations lying in the immediate path of any lava flow.

As described above, well-established response protocols to mitigate effusive risk at Piton de la Fournaise have thus been in place since 1979, and widely reported in the scientific literature. There are a vast number of papers in the English language (peer-reviewed) scientific literature dealing with lava flow hazards, effects and impacts of lava flow events at Piton de la Fournaise (Stieltjes and Moutou 1989; Delorme et al. 1989; Villeneuve and Bachèlery 2006; Bhugwant et al. 2009; Michon et al. 2013; Tulet and Villeneuve 2011; Gouhier and Coppola 2011), as well as monitoring (e.g. Peltier et al. 2009, 2018, 2020; Roult et al. 2012), response and mitigation (Harris et al. 2017a, 2019; Peltier et al. 2020) measures implemented during effusive crises at this frequently active effusive center. Additionally, over a 42-year period, thousands of bulletins and reports have also been issued by OVPF-IPGP and communicated to the *Préfecture,* the media and the public at large. These detailed OVPF-IPGP reports are sent if there is any change in activity, both during eruptive and non-eruptive periods. During eruptive periods they are issued daily to all stakeholders to aid in assessing the risks associated with lava flow crises. They are posted and archived on the OVPF-IPGP website (http://www.ipgp.fr/fr/ovpf/actualites-ovpf) where they are openly available. Reports are also distributed by various social media (Twitter, Facebook) as well as email distribution lists. Reports are mostly in French because releases need to be used by local civil protection, media and population, and so have to be in the home-country language. These reports are complemented by automatic daily bulletins and detailed monthly reports. The monthly reports have also been available in English since 2018 (ISSN 2610–5101; http://www.ipgp.fr/fr/dernieres-actualites/344).

## Constructing the gap analysis database: damaging eruptions since 1950

Since 1950 (i.e., the starting date of the Tsang and Lindsay (2020) gap analysis), eruptions at Piton de la Fournaise have been dominantly effusive, with Hawaiian to Strombolian style activity at the source fissures. Lava flows are generally confined to the Enclos caldera (Fig. 1). This structure is a 13 × 8 km amphitheater-shaped caldera open to the east. The caldera contains a terminal shield, topped by the Cratère Dolomieu, which is the main centre of activity. This amphitheatre structure is extremely important in terms of hazard as, like the Sciara del Fuoco at Stromboli (cf. Barberi et al. 1993; Calvari et al. 2011) it is uninhabited but the caldera walls and slope to the east contains lava flow to within its limits, and delivers lava relatively harmlessly towards the ocean. Thus, although lava flow hazard occurrence inside the caldera is extremely high, risk to population is close to zero. However, risk to hikers (of which there are more than one hundred thousand each year; Derrien et al. 2019; Villeneuve 2020) is considerably higher, as the main access paths cross active rift zones and paths of frequent lava flow inundation (Fig. 1). A relatively important risk concerns the island belt road (RN2), which crosses the lower part of the Enclos at an elevation of about 100 m above sea level. The exposed section is about 9.5 km long (Fig. 1). An average of 4530 vehicles per day were counted as using this road segment in 2014 [INSEE (2014) in *Tableau Economique de La Réunion* – www.insee.fr; Villeneuve 2020]. Termed “*Hors Enclos*” (*hor*s meaning outside) events, it is the rarer eruptions fed by dykes that pass beyond the limits of the caldera to feed events beyond the Enclos that pose a risk to populations living on the flanks of the volcano (cf. Fig. 1b).

A public report (in French), published in 2012, evaluated volcanic hazards at Piton de la Fournaise, and presented a comprehensive lava flow hazard map (Di Muro et al. 2012; Davoine and Saint-Marc 2016). This hazard map has recently been updated by Chevrel et al. (2021). Indeed, for all eruptions since 1931, lava flow inundation areas during all eruptions have been mapped to allow generation of an exhaustive database for lava flow coverage (Derrien 2019; Chevrel et al. 2021). We see that, since 1950 (i.e., the starting date of the gap analysis as for Tsang and Lindsay 2020), only three eruptions have occurred in inhabited areas outside of the Enclos (Fig. 1c), these being in April 1977, March–April 1986 and August–September 1998 (Stieltjes and Moutou 1989; Lénat and Bachèlery 1988, 1990; Lénat et al. 1989, 2012; Villeneuve and Bachèlery 2006; Peltier et al. 2009; Tanguy et al. 2011; Michon et al. 2013, 2015). Of these three *Hors Enclos* eruptions, only two (April 1977, March–April 1986) were associated with lava flows that threatened populated areas, impacting towns located more than 10 km from the summit and leading to evacuation (Stieltjes and Moutou 1989; Villeneuve and Bachèlery 2006; Harris and Villeneuve 2018a, b).

Between 1950 and 2020, eight eruptions within the Enclos (April 1961, January 1976, June–July 2001, January 2002, November–December 2002, August–October 2004, February 2005, March–May 2007; Fig. 1c) caused damage to public infrastructure by crossing RN2 (e.g., Roult et al. 2012; Michon et al. 2013; Rhéty et al. 2017; Harris and Villeneuve 2018a, b). Other eruptions, such as those of May 2015 and April–May 2018, required evacuation of observatory equipment (e.g., monitoring stations and transmission relays) that lay in the paths of active lava flows (Harris et al. 2017a, 2019). However, all of these events occurred inside the uninhabited caldera, and so did not cause damage to population centres.

Populations outside of the Enclos were also evacuated during the January 2002 eruption due to the threat of fissures opening outside of the caldera at the end of the eruption (Villeneuve and Bachèlery 2006). In addition, an evacuation occurred during the March–May 2007 eruption due to fake news reported in the local media of the opening of an eruptive fissure outside of the Enclos and above the village of Le Tremblet (Morin 2012; Harris and Villeneuve 2018a, b). In April 2007, some of the population experienced health problems due the high concentrations of sulfur dioxide; and at-least one school had to be evacuated (Harris and Villeneuve 2018a, b). In summary, evacuations occurred in April 1977, March 1986, January 2002 and April 2007. For all eruptions where lava flows crossed RN2 within the Enclos, the road was closed on either side of the lava crossing point by local authorities a few hours before its inundation (cf. Harris and Villeneuve 2018a, b).

Based on this literature, we provide a complete and correct database for all eruptions that have threatened inhabited areas or infrastructures since 1950 at Piton de la Fournaise in Table 1. This is a revised version of the Table “*summarising basaltic lava flow events at Piton de la Fournaise Volcano that have threatened inhabited areas since 1950*” of Tsang and Lindsay (2020). The Table of Tsang and Lindsay (2020) considers only a very few of the sources cited here, and includes a number of errors and mis-representation of those sources that are cited. We address these issues, point-by-point, in the Electronic Supplementary Material. In this regard, it is exceedingly important to ensure that  this database is complete and correct as it serves the basis for the ensuing gap analysis, so if it is incomplete or incorrect it will bias the outcome.

**Table 1:** Table summarizing information from reports of basaltic lava flow events at Piton de la Fournaise that have threatened inhabited areas or infrastructures since 1950

Eruption	References	Overview, including impacts (Eruption duration)	Response	Recovery & Applying lessons learned
**1961 (Apr.)**	e.g. Derrien 2019; various local newspapers	Lava flows inundated the coastal highway. (61 days)	A section of the coastal highway was closed	Part of the coastal highway was rebuilt
**1976 (Jan.)**	e.g. Derrien 2019; various local newspapers	Lava flows inundated the coastal highway. (4 months)	A section of the coastal highway was closed	Part of the coastal highway was rebuilt
**1977 (Apr.)**	e.g. Tricot and Vincent 1977; Kieffer et al. 1977; Davoine and Saint-Marc 2016; various local newspapers	Lava flows threatened the town of Bois Blanc and inundated thirty structures in Piton Sainte Rose. A main road and bridge were also inundated. (11 days)	More than 1000 people were evacuated	This eruption prompted the creation of the volcanic observatory in 1979
**1986 (Mar.)**	e.g. Bertile, 1987; Delorme et al., 1989; Davoine and Saint-Marc, 2016; OVPF bulletins; various local newspapers	This eruption consisted of four phases. During the second phase, the coastal highway was traversed by two lava flows. Eight rural houses were destroyed. During the third phase, steaming cracks opened across the coastal highway and a lava tube formed across the coastal highway. (9 days)	Towns were evacuated. Fifty-one people were made homeless by the destruction of their homes	Part of the coastal highway was rebuilt
**1998 (Mar. - Sep.)**	e.g. Villeneuve et al 2008; Peltier et al. 2009; Di Muro et al. 2012, 2014; Davoine and Saint-Marc 2016; Staudacher et al. 2016; Vlastélic et al. 2018; OVPF bulletins; various local newspapers	A lava flow threatened Bois Blanc but did not reach the town. Another lava flow stopped 5 m from the coastal highway. Forest fires were ignited. (6.5 months)	A section of the coastal highway was closed	
**2001 (Jun. - Jul.)**	e.g. Villeneuve et al 2008; Peltier et al. 2009; Di Muro et al. 2012, 2014; Davoine and Saint-Marc 2016; Staudacher et al. 2016; Vlastélic et al. 2018; OVPF bulletins; various local newspapers	Two lava flows inundated the coastal highway. (3.7 weeks)	A section of the coastal highway was closed	Part of the coastal highway was rebuilt
**2002 (Jan.)**	e.g. Villeneuve et al 2008; Peltier et al. 2009; Di Muro et al. 2012, 2014; Davoine and Saint-Marc 2016; Staudacher et al. 2016; Vlastélic et al. 2018; OVPF bulletins; various local newspapers	A lava flow cuts the coastal highway before creating an ocean entry. A lava delta of 15 ha formed. (1.5 weeks)	A section of the coastal highway was closed	Part of the coastal highway was rebuilt
**2002 (Nov. - Dec.)**	e.g. Villeneuve et al 2008; Peltier et al. 2009; Di Muro et al. 2012, 2014; Davoine and Saint-Marc 2016; Staudacher et al. 2016; Vlastélic et al. 2018; OVPF bulletins; various local newspapers	A lava flow created an ocean entry after crossing the coastal highway. (2.8 weeks)	A section of the coastal highway was closed	Part of the coastal highway was rebuilt
**2004 (Aug. -Oct.)**	e.g. Villeneuve et al 2008; Peltier et al. 2009; Di Muro et al. 2012, 2014; Davoine and Saint-Marc 2016; Staudacher et al. 2016; Vlastélic et al. 2018; OVPF bulletins; various local newspapers	A lava delta was created after a lava flow cut the coastal highway. (8.8 weeks)	A section of the coastal highway was closed	Part of the coastal highway was rebuilt. The lava tubes are now a tourist attraction.
**2005 (Feb.)**	e.g. Villeneuve et al 2008; Peltier et al. 2009; Di Muro et al. 2012, 2014; Davoine and Saint-Marc 2016; Staudacher et al. 2016; Vlastélic et al. 2018; OVPF bulletins; various local newspapers	Two lava flows inundated the coastal road. (1.3 weeks)	A section of the coastal highway was closed	Part of the coastal highway was rebuilt
**2007 (Mar. -May)**	e.g. Payet et al. 2007; Bhugwant et al. 2009; Peltier et al. 2009; Viane et al 2009; Staudacher et al. 2009, 2016; Bertile 2011; Morin 2012; Di Muro et al. 2012, 2014; Davoine and Saint-Marc 2016; Rhéty et al., 2017; Harris and Villeneuve 2018a, 2018b; Vlastélic et al. 2018; OVPF bulletins; various local newspapers	A lava flow buried the coastal highway (eventually under 50 m of lava) before reaching the ocean. Gas emission caused health issues. The resulting laze damaged metal roofs within 1 km of the ocean entry. By the end of the eruption, 1.4 km of the coastal highway were inundated while 0.45 km^2^ of new land was created. This was one of the most voluminous eruptions in centuries, damaging forests and igniting fires; causing vegetation damage as far away as Mauritius. The ocean entry impacted sea life with dead fish floating up from depths down to 500. The local fishing industry was severly impacted (1 month)	A section of the coastal highway was also closed. The town of Le Tremblet was evacuated after fake news announcing an "hors-Enclos" fissure opening. Three schools were evacuated and medical aid given due to gas impacts. Warnings/advice were issued (via the local newspaper) for farmers, aviation and those approaching the ocean entry.	Part of the coastal highway was replaced within a few months (cost: 1 million €). Viewing points were set up for sight-seer access

It is also important to state the limits of the literature search, and rules for literature and event to be counted for inclusion in the Table. Here, we only consider effusive events that threatened the population and inhabited areas and infrastructure, including RN2, public land, plus internet, telephone and electric networks. We omit cases of forest fire, hiking trail damage, evacuation of observatory equipment and visitor accidents. However, we do note that since 1950 and through September 2021, 25 people have died on the volcano, with 40% of cases being recorded during an eruption (although only three was directly due to lava flow contact; others being linked to mountain accidents, as people who get lost or who fell from the cliff). In addition there are around 58 helicopter evacuations per year due to injuries (average between 2001 and 2015), although again these are typically visitor accidents and not necessarily during an eruption. With these caveats we consider all events between 1950 and 2020 (Fig. 1), and search all peer-reviewed literature in French and English. Also, where necessary, we consider observatory reports and newspaper reporting, which are generally (and by necessity) in the home language, i.e., French (see notes in the Electronic Supplementary Material for the potential effect of not using this resource). This can now serve as a basis for a valid gap analysis for effusive eruptions at Piton de la Fournaise in terms of preparedness, response and recovery, which we complete next.

## Piton de la Fournaise: preparedness, response and recovery gap analysis

Our gap analysis for Piton de la Fournaise is given in Table 2. We complete the analysis based on the same criteria and categories as used by Tsang and Lindsay (2020), but using our database of Table 1 and providing modifications and clarification of the scope of each category.

Category: *Preparation actions and narrative*

Criteria: This category includes all published monitoring data (i.e., descriptions of preparation actions through installation and use of monitoring networks) as well as published narratives of eruptive events. This considers data in papers published in the peer-reviewed literature (in English and French) as well as in publically-available reports on institutional websites. It includes descriptions of preparation actions undertaken prior to eruption onset, as well as use of monitoring data acquired during eruptions to allow situation updates.

**Table 2:** Table indicating the research gaps identified (−) and whether data for each research theme have been collected and published (✓). See Table 1 for references

Eruption	Preparedness		Response						Recovery		
	Preparatory narrative	Lava flow hazard modelling	Eruption narrative	Lava flow attributes	Detail of physical impact data	Response narrative	Communication approach	Evacuation data*	Recovery (including abandonment of land) narrative*	Community reactions*	Application of experience / “lessons learned”
**1961 (Apr.)**	−	−	✓	✓	−	−	✓	n.a.	n.a.	n.a.	−
**1976 (Jan.)**	−	−	✓	✓	−	−	✓	n.a.	n.a.	n.a.	−
**1977 (Apr.)**	✓	−	✓	✓	✓	✓	✓	✓	✓	−	✓
**1986 (Mar.)**	✓	−	✓	✓	✓	✓	✓	✓	✓	✓	✓
**1998 (Mar. - Sep.)**	✓	−	✓	✓	✓	✓	✓	n.a.	n.a.	n.a.	✓
**2001 (Jun. - Jul.)**	✓	−	✓	✓	✓	✓	✓	n.a.	n.a.	n.a.	−
**2002 (Jan.)**	✓	−	✓	✓	✓	✓	✓	✓	−	✓	−
**2002 (Nov. - Dec.)**	✓	−	✓	✓	✓	✓	✓	n.a.	n.a.	n.a.	−
**2004 (Aug. - Dec.)**	✓	−	✓	✓	✓	✓	✓	n.a.	n.a.	n.a.	✓
**2005 (Feb)**	✓	−	✓	✓	✓	✓	✓	n.a.	n.a.	n.a.	✓
**2007 (Mar. - May)**	✓	✓	✓	✓	✓	✓	✓	✓	✓	✓	✓

n.a.: not applicable; no evacuation of residents: but access to uninhabited areas and part of the RN2 road closed

* Only considering evacuation of inhabitants. Note that hikers are always evacuated from the Enclos, which is an area to which access is prohibited during eruptions

As described above, the OVPF-IPGP monitoring network has been operational since 1979, and there is a huge database of reporting material. The presence of these documents means that all eruptions after 1979 have been well-documented (i.e. in the first column of Table 2 are all checked). Additionally, Kieffer et al. (1977) and Tricot and Vincent (1977) provided data for the 1977 eruption (which is therefore also checked in Table 2).

Category: *Lava flow hazard modelling*

Criteria: This category includes all types of lava flow hazard modelling both prior or during an eruption. We also add studies intended to initialize lava flow models with appropriate source terms so that they are initialized, and ready for use, during the next crisis.

In addition to the lava flow hazard maps published by Di Muro et al. (2012) and Chevrel et al. (2021), a response protocol is in place that involves real-time model-based assessment of likely lava flow inundation areas. This has been in place since 2014 (Harris et al. 2017a, 2019), and has been implemented for all eruptions since that of June 2014. This involves delivery maps showing likely lava flow inundation areas to Civil Protection (Peltier et al. 2020). All eruptions listed in Table 2 are prior 2007 and therefore cannot be checked. However, if we consider lava flow modelling for preparation and initialization of models aimed at simulating flow emplacement dynamics and run out (to be used for probabilistic risk maps and near-real time simulations), then we should fill the gap for the 2007 eruption. For this “paroxysmal” (high intensity) event, Rhéty et al. (2017) completed thermo-rheological modelling so as to allow initialization of the FLOWGO simulation (Harris and Rowland 2001) for this event type.

Category: *Eruption narrative*

Criteria: This category includes all documents giving an overview description of the eruption.

As detailed above, daily bulletins are systematically sent to local authorities by OVPF-IPGP and have been published on the observatory website since 2014. Even for the pre-1979 eruptions (before the existence of the observatory), there is extensive documentation and reporting on the activity in a variety documents: see Michon et al. (2013) and Derrien et al. (2019) for a compilation.

In terms of hazard narrative, the lava flow hazard map for Piton de la Fournaise is based on all mapped historical events (Di Muro et al. 2012; Davoine and Saint-Marc 2016); events which are also detailed in Nave et al. (2016). In addition, Michon et al. (2013) analyzed all historical texts concerning eruptive activity on Piton de la Fournaise. These texts often refer to notes about volcanic activity written to inform those in charge of the colony, a practice which has been regular since the 17th century. Dupéré (personnal communication) also notes that high school teachers have been funded to archive volcanic phenomena since the 19th century and that, very early in the history of recreational aviation in La Réunion, pilots informed on the occurrence of volcanic activity. In addition, the local newspapers are a rich source of reliable and detailed narrative (cf. Harris and Villeneuve 2018a, b). Thus, this box is checked for all cases in Table 2 so that we find no knowledge gaps.

Category: *Lava flow attributes*

Criteria: This category refers to any reports of lava flow observations and/or measurements, for example of lava flow flow front advance rate, velocities, effusion rates, temperature, texture, geochemistry…).

Since the creation of OVPF-IPGP, all lava flows have been systematically sampled during (or shortly after) all eruptions. Additionally, all historical lava flows have been sampled (as listed in Vlastélic et al. 2018). Often multiple samples are available as time series for an eruption. These samples are quickly and routinely measured to obtain the chemistry and texture of the lavas, and have served the basis for many publications (e.g., Vlastélic et al. 2007; Villeneuve et al. 2008; Peltier et al. 2009; Di Muro et al. 2014; Rhéty et al. 2017; Harris et al. 2020). The analyses are made publically available (in English) on the DynVolc portal from Observatoire de Physique du Globe de Clermont Ferrand (http://wwwobs.univ-bpclermont.fr/SO/televolc/dynvolc/bdd.php; Gurioli et al. 2018), and used to initialize lava flow simulations for risk appraisal (Harris et al. 2017a, 2019). Thus all eruptions are checked for the “attributes” category in Table 2.

In addition, lava flow front locations and propagation rates are described in daily eruptive bulletins, where Harris et al. (2019) explain how the flow contours are obtained from, amongst other methods, satellite or airborne images to allow tracking of flow field expansion. These, and other InSAR-derived attributes are available from the Observatoire InSAR de l’Océan Indien (Richter and Froger 2020: https://opgc.uca.fr/volcanologie/oi2). Finally, time averaged discharge rate times series are available for Piton de la Fournaise from satellite-base monitoring systems: MIROVA (Coppola et al. 2016: https://www.mirovaweb.it/) and HOTVOLC (Gouhier et al. 2016: https://hotvolc.opgc.fr/www/index.php), as well as MODVOLC (Wright et al. 2002: http://modis.higp.hawaii.edu/). These provide spectral radiance and TADR attributes for all eruptions back to 2000, or all eruptions back to 1979 using Advanced Very High Resolution Radiometer (AVHRR) data (cf. Harris et al. 2011), and are used routinely during effusive events to provide update of this attribute (e.g., Coppola et al. 2017; Harris et al. 2017a; Peltier et al. 2020). Since 2010, lava effusion rates have also been calculated from SO_2_ fluxes following the procedure described in Hibert et al. (2015).

Category: *Detail of physical impact data*

Criteria: This category should encompass analysis of damage, including surveys of structures, their properties and degree of damage, as well as the availability of databases for structure types and construction materials.

Systematic aerial reconnaissance has been carried out during every eruption since 1979 by the Gendarmerie (one of the two police forces in France). Overflights are carried out in consultation with OVPF-IPGP to assess damage and damage potential, and these observations are published in the daily reports. Overflights are also crewed after each eruption by the ONF (Office National des Forêts, the French national forestry office), which is the land manager responsible for the hiking trails. Also on-board are the PGHM (Pelotons de Gendarmerie de Haute Montagne – the gendarmerie group responsible for high mountain areas), which is responsible for search and rescue in the case of mountain accidents. Additionally, a member of the BRGM (Bureau de Recherches Géologiques et Minières – the French national office of geological and mining research), whose role is to assess the condition of the caldera walls near which hikers pass, is present; as well as an OVPF-IPGP staff member. All observations of damage are thus well documented, so that this column is checked in Table 2 for all eruptions after 1979.

Detailed data of what and how the built environment sustained damage (e.g., Jenkins et al. 2017), is also available from documented case, such as the 1977 eruption (Vaxelaire 2012) during which stone buildings (including the church) were damaged but survived; however, all the wooden structures were burnt. Likewise, maps exist for lengths of roads, trails and trail markers destroyed (e.g., Fig. 1), as well as for lava flow thickness (see: Bato et al. 2016; Derrien 2019). This allows structures to be placed into a hazard GIS, as done by Di Muro et al. (2012), and correlations with lava flow attributes such as lava flow thickness; a step essential to determine the hazard posed to, for example, buried infrastructure such as electricity, gas, telephone and water networks.

Category: *Response narrative*

Criteria: This category refers to description of the response actions taken because of an eruption

All eruptions since 1977 have led to response actions, which have been thoroughly reported, described and documented. We note that OVPF-IPGP was created in response to the 1977 eruption and, since then, detailed daily bulletins in French (ISSN 2610–5101), supported by a monthly review in French and English, have been issues prior to, during and after every eruption. These are sent to the local authorities, distributed via social media and email distribution lists, and published (i.e., made publically available) by posting on the OVPF-IPGP website (e.g., https://www.ipgp.fr/fr/dernieres-actualites/344).

Category: *Communication approaches*

Criteria: This category refers to published accounts of a communication approach or newspaper articles quoting scientists or emergency managers.

All eruptions at Piton de le Fournaise are subject to abundant reporting in the local and national (and sometimes international) press and media outlets. Many of these articles quote scientists, emergency managers and/or clarify communication of the alerting system (e.g., Flash actu, Le Figaro 2016). Based on an analysis of reporting during the 2007 eruption, Harris and Villeneuve (2018a, b) concluded that one local island newspaper, the Journal de l’Ile de la Réunion (JIR), is a quality information source (for all eruptions) and that the source of information is always appropriate and correct. Harris and Villeneuve (2018a, b) concluded that the JIR is of a high educational value and effective in transmitting information related to volcanic hazard, response and for archiving the event narratives. Every eruption since 1979 has involved at-least one newspaper report quoting scientists (usually OVPF-IPGP) and emergency managers, and when necessary describing the communication approach. The communication approach, call down procedure and collaborative network for an effusive crisis response is also given in Harris et al. (2017a, b), as well as in Peltier et al. (2020) and the government-mandated ORSEC (Organisation de la Réponse de Sécurité Civile) response plan (https://www.gouvernement.fr/risques/dispositif-orsec). This category therefore has no gap, and even for the 3 eruptions prior 1979 as the newspaper articles were abundant (Table 2).

Category: *Evacuation data*

Criteria: This category includes all published information about whether an evacuation occurred.

For this category, it is important to decipher whether we consider only the evacuation of the population living on the volcano flanks, or also evacuation of hikers from the Enclos. As well as spontaneous versus officially declared and managed evacuations. Resident populations have been evacuated only in April 1977, March 1986, January 2002 and April 2007, the last case being a “spontaneous” evacuation following fake (or confused) news (Harris and Villeneuve 2018a, b). Indeed in 2007 evacuation was never officially declared by the Prefecture or the Mayor’s office, but was instead implemented on the ground by the responders in-place who panicked as they, themselves, were frightened and unsure (Julie Morin, personal communication); as well as those residents who fled a frightening/stressful situation of their own accord (JIR 2007).

But for all eruptions, if hikers are present during the seismic crisis preceding the eruption (Roult et al. 2012; Peltier et al. 2018), they are immediately evacuated from the Enclos by the *Gendarmerie* (this being a requirement of the Alert 1 level of the ORSEC plan) and access to the summit is closed. Thus, to list an event on the basis of “evacuation” and/or “closure” would mean listing all eruptions, and the list would become meaningless. We thus only consider here the cases for which the population living on the volcano was evacuated (spontaneously or officially).

Category: *Recovery narrativ*e

Criteria: This category refers to publically-available narratives giving recovery actions, and timeline of recovery actions needed. We only considered here recovery actions after evacuation of inhabitants, and disregarded recovery actions to rebuild RN2 each time it is cut and/or to reopen hiking routes after each eruption, which are always reported in the newspaper, as shown by Harris and Villeneuve (2018a, b).

There are instructive recovery narratives for both eruptions that entered populated areas in 1977 and 1986, for example: Bertile (1987) and Vaxelaire (2002); as well as for the 2007 eruption (Bertile 2011; Morin 2012). The other eruptions did not require evacuations, except 2002, so there are no other narratives. Note that all of these sources, especially the detailed and meticulous debrief of Bertile (1987) and of Payet et al. (2007) are in French.

Category: *Community reactions*

Criteria: This category refers to the reaction of the community to actions taken during the implementation of emergency management efforts during the recovery phase following an evacuation.

The community reactions during the recovery phase for the March 1986 eruption are described in Bertile (1987), and community reactions for the 2002 and 2007 eruptions are reported in Morin (2012), as well as in Payet et al. (2007) and Harris and Villeneuve (2018a) for 2007. In 2007, the inhabitants returned to their houses once it was considered safe to do so (Morin 2012). Additionally, in 2002 (but also in 2007), several inhabitants, through fear of being robbed while their houses were abandoned, refused to leave their homes (Imazpress 2002).

Category: *Application of experience / “lessons learned”*

Criteria: This category includes the documented actions (in the scientific literature) taken after an eruption that could help for the next eruption.

The first eruption for which the application of the “lessons learned” applies is the 1977 eruption that resulted in the establishment of the OVPF-IPGP 2 years later. In the analyses of Morin (2012), the improvements made by the scientists and local authorities after the 1986 and the 2007 eruptions are also described. Applications of learning have also been made for the 1998, 2004 and 2005 eruptions, as stated in the *PSS Volcan: Plan de Secours Spécialisé Volcan “Piton de la Fournaise”* as drafted by the *Préfecture de la Réunion* in 2005. This document describes the protocol to access the Enclos and the rules for management of large gatherings (e.g., of sightseers) and traffic management. This document also handles the case the RN2 being cut by lava (as based on the lessons learned from the eruptions of August 2004 and February 2005), which isolates the population on the southern end of the island. It also handles cases when the eruption is visible from the Pas de Bellecombe car park (Fig. 1) following lessons learned from the eruptions of March 1998).

Applications of learning were also made after the 2007 eruption, after which there was, for example, an the enhancement of the network dedicated to monitor volcanic air pollution on the island (by *ORA: Observatoire Réunionnais de l’Air*) and the deployment of emergency units for medico-psychological help (*CUMP: Cellules d’urgence médico-psychologique*). The documents reporting these lessons learned are not always public. However, these are available upon request to the observatory or authorities responsible for contracting the reports and plans. To compile exhaustive, accurate and useful information of this type it is therefore necessary to refer to an expert of the site considered (ideally the Scientist in Charge of the responsible observatory). We therefore recommend that this category be clarified so that it includes “documentation upon request” to take into account publically available documentation that is not necessarily available in the peer-reviewed literature. Thus, the “in the scientific literature” caveat and requirement of Tsang and Lindsay (2020) excludes a significant body of work and formal reporting by observatories and partner agencies and thus needs to be added to this criterion. For the analysis here, though, this category was only checked for the 1977 eruption because the OVPF-IPGP was built after this eruption, and this is well described in the literature (e.g. Peltier et al. 2020); but for the other cases there was no public information. But if we take into account our caveat, we could also check 1986, 1998, 2004, 2005 and 2007.

## Discussion

The result of our adjusted gap analysis, which involves a survey of a complete review of all relevant sources reveals that, for Piton de la Fournaise, there are very few gaps in the ability of OVPF-IPGP to monitor, respond and mitigate during effusive crises. Eruption responses rely on well establish protocols and trust between observatory staff, emergency managers, and the public, with systematic feedback between each actor after each eruption. This feedback has resulted in a high degree of application of experience and “lessons” (Table 1), resulting in well-developed capability with few gaps (Table 2).

### Lava flow hazard modelling

In terms of modelling lava flow emplacement dynamics and run out for Piton de la Fournaise, our gap analysis reveal that no modelling existed prior to the events listed in Tables 1 and 2. In addition, no flow was modelled during the crises. This is the main gap, where the selected eruptions in Table 2 show a wide gap in this category. An exception, though, is the 2007 eruption for which subsequent work by Rhéty et al. (2017) focused on thermo-rheological modelling so as to initialize FLOWGO (Harris and Rowland 2001) to this event. Recognizing this gap, the FLOWGO model has been initialized for more typical effusive events using the 2010 eruption (Harris et al. 2016), and further developed by Chevrel et al. (2018) so as to be easily executable for effusive events at Piton de la Fournaise through provision of the open-source code PyFLOWGO. As a result it is now used operationally to make model-based hazard assessments for any new effusive event (Peltier et al. 2020). Since 2014, this has been embraced as part of an effusive crisis response protocol (Harris et al. 2017a, 2019), and contributed to the update of the long term lava flow hazard map (Chevrel et al. 2021). This has resulted in generation of a GIS (Geographic Information System) based on lava flow modelling below which there are layers including vulnerable structures and highways to allow risk assessment (cf. Latutrie et al. 2016). This protocol allows projections of potential inundation zones to be made using DOWNFLOW (Favalli et al. 2005) and PyFLOWGO (Chevrel et al. 2018) in the first hours (sometimes minutes) of the start of an eruption (Peltier et al. 2020). Since 2019, inundation potential maps, including the conditions necessary to cut RN2, have been delivered to authorities and provide support for decision responses (Peltier et al. 2020). Thus the gap analysis of Table 2 has been applied, and used to fill a recognized gap in this category.

### Recovery categories

The three recovery categories in Table 2 also have a high number of cases for which the gap analysis is not applicable, i.e., "n.a.". This is because most eruptions did not directly affect the inhabitants, except for the three events in 1977, 1986, and 2007, for which all categories are well documented. The lack of documentation in the scientific literature is therefore, in these cases, simply linked to the fact that only the RN2 was threatened by lava flows and therefore closed to traffic. For all cases when the RN2 has been cut, the road has been rebuilt generally within a few weeks/months following the end of each eruption. The second reason, is the limit placed on considering “scientific literature” or the “peer reviewed” literature, and limiting this search further by only considering English language publications (cf. Tsang and Lindsay 2020). Such events and the actions taken by stakeholders such as government bodies and emergency managers in repairing or replacing critical infrastructure, tend not to be covered in scientific work. Instead, it is the domain (as argued above) of observatory reporting and newspaper reporting. We thus address the issue of the appropriate scope of a gap analysis next.

### Scope of the gap analysis in terms of literature search

Limiting a gap analysis focused on volcanic hazard, risk and mitigation to a consideration of the English language scientific literature will severely impede the completeness of the analysis. This is especially true when considering a case in a non-English speaking country. The local press is a particularly rich source of information, where preparation measures, impacts, and recovery are eminently newsworthy and thus usually reported, often extensively (Harris et al. 2012). Local newspapers are always written in the home language, but archives are commonly accessible via the internet with back issues available. The media is thus a source that can be easily used, and will likely contain far more data than the scientific literature. For example, Blong (1984) in his exhaustive source book on volcanic hazard draws heavily on newspaper reports, as does Sterling (1997) in his book on weather hazard in Britain. In fact, the chronology of weather hazard in France by Séchet (2004) draws entirely on newspaper reports. Thus, in Table 2, although the scientific literature is quite complete for all events considered, we also turn to the local newspapers to provide information on, and check for gaps in, our state of knowledge for impacts, response and recovery. For the island of La Réunion there are two main newspapers, Le Journal de L’Ile de La Réunion and Le Quotidien, whose archives are available (in French) on https://www.clicanoo.re/ and http://www.lequotidien.re/, respectively. The archives of Le Journal de L’Ile de La Réunion, in particular, have been shown to contain a wealth of data for the impacts of, response to and recovery from effusive events at Piton de la Fournaise (Harris and Villeneuve 2018a, b).

The non-scientific literature is also an immense resource to support a gap analysis focusing on hazard and risk, where there is a vast amount of information in published family histories, travel diaries and/or local histories. For the Vulcano’s 1888–1890 eruption, for example, Hew Stevenson’s history of the Stevenson family provides information from a letter written by James Stevenson, who at the time owned the island (Stevenson 2009). This can be used to document the damage observed and injuries recorded on Vulcano during the eruption, as well as the response, where the island was sold and the Stevenson family left, their home having been destroyed by bomb impact. As another example, the diary of William Ellis (Ellis 1825) is a well-known source of information for volcanic features on the island of Hawaii, but also records damage inflicted by, and response to, Kïlauea’s effusive eruption of 1859. For La Réunion, volume 2 of Daniel Vaxelaire’s popular history of La Réunion (Vaxelaire 2012) records damage inflicted on the town of Piton Sainte Rose due to lava ingress during the April 1977 eruption of Piton de la Fournaise.

This latter source is also in French, highlighting the need for a multi-lingual search at target sites that are non-English speaking. However, this guideline may also be applied to a search focusing solely on the scientific literature to assess gaps purely in research. Many scientific articles regarding volcanic hazard in France, Italy, Iceland, Russia and Japan, for example, are available but in journals and books that are published only in the home language (Harris et al. 2017b). As a result, because the potential database is so large, to avoid gaps in the gap analysis itself the search needs to be as rigorous as possible ensuring that all possible scientific literature has been considered, in all languages and disciplines, and potentially including popular literature and the media if the focus is damage, response and recovery. This may highlight a need to target a single site or case (as done here) so that the analysis can be as broad (in terms of type of literature considered), extensive, exhaustive, complete and, thus, as representative of the state-of-knowledge as possible. Thus, some sources listed in Table 1 are in French (e.g., Tricot and Vincent 1977; Bertile 1987; Villeneuve and Bachèlery 2006) and from non-volcanological sources, where we note that Viane et al. (2009) is in the French language scientific journal devoted to allergies.

### Preparatory and recovery actions: “learning”

When considering preparatory and recovery actions, several questions can be raised regarding “memory”. For example:

- What time limit do you place on how long after a particular eruption “learning” outcomes are still being implemented?
- How long do we have to wait before we can consider that a preparatory action is related to a specific eruption, or a series of experiences (especially at a frequently active volcano such as Piton de la Fournaise)?
- How long a time after an eruption can we consider that a recovery action has been completed, and that it is still being “learned from” and the “lesson applied”?

Defining whether a lesson is learned and applied is not an easy task, and raises further questions. For example, as considered above for the “Application of experience” category, how do we quantify a process that is not necessarily reported in the literature when it is actually widely applied and documented in a variety of ways on the field? The solution is to expand the scope of the analysis and criteria as recommended above.

Each eruption is a new experience for all actors involved and contributes to building up their background knowledge of the phenomenon in the long term. As a result, this is an exceeding ambiguous category because an observatory goes through a learning exercise during and after every eruption, and is increasing its knowledge and applying lessons continually (cf. Driedger et al. 2020). At Piton de la Fournaise some key steps in the learning process, and advances due to application of knowledge and experience, were, for instance:

- 1980: First deformation network installed on the volcano (Blum et al. 1981);
- 1981: First eruption anticipated at Piton de la Fournaise by the early set up seismic network (Bachèlery et al. 1982);
- 1984: First volume calculation by photogrammetry (Delorme 1994)
- 1998: First location of tremor source during “Hors Enclos” magma propagation, and first relation defined between tremor and eruption rate (Battaglia et al. 2005);
- 1998: First ground deformation measured by RadarSat1 interferogram (Sigmundsson et al. 1999)
- 1998: First ground deformation measured by GPS (Briole in Villeneuve 2000)
- 1999: First use of hand-held camera imagery (Staudacher et al. 2000);
- 2003: First interferograms using ASAR sensor on ENVISAT satellite (Froger et al. 2004)

So, in effect, if a paper is published following an eruption: this is a learning action. Thus, we either need to check the “Application of experience” box in Table 2 whenever a paper is published, or caveat by stating:

“This category includes documented actions in the public domain that led to changes in public and/or observatory policy, response actions, mitigation protocols, installation and use of monitoring arrays, and/or disaster management plans”.

This, really is, a question that only on-site actors charged with disaster response duties can answer.

### Problems arising from poorly implemented gap analyses

Our results are in contrast to the result of the Tsang and Lindsay (2020) for Piton de la Fournaise because the information considered for Piton de la Fournaise was incomplete and in error (see Electronic Supplementary Material) and, in many cases, the criteria poorly or inappropriately defined. As a result, we find the gap analysis of Tsang and Lindsay (2020) for Piton de la Fournaise to be invalid. Unfortunately, such a poorly-informed and erroneous, database — and hence conclusion regarding “gaps”, is detrimental to the operations of the host volcano observatory. It is also potentially erosive of the confidence that all actors involved in crisis response (including the impacted population) have in the body charged with monitoring and responding to any crisis or with drawing up risk assessment and hazard response protocols. In the case analysed here, this is the OVPF-IPGP, as well as the response plan itself.

## Conclusion

The failure of the gap analysis of Tsang and Lindsay (2020) for Piton de la Fournaise was based on an inappropriate methodological approach that did not consider all the available literature or, indeed, key literature. A more robust approach, as we provide here, involves recourse to observatory reports, newspaper articles, and interviews with stakeholders and observatory directors, as well as a proper consideration of the full scientific literature. Blong (1984), for example, relies in a large part on newspaper sources for his seminal source book on volcanic hazard. We note that, instead, Tsang and Lindsay (2020) relied, in a large part, on the Global Volcanism Program (GVP) database, ignoring that although Pallister et al. (2019) argued that

“added value (to risk assessment) can be attained through analysis of global databases on past eruptions”

such as GVP, they caution that

“one must be aware of the limitations, biases and default values that are inherent in such databases”.

Pallister et al. (2019) also add that such databases

“lack precursory and eruption phase details and monitoring data”.

We find this problem of bias here in the analysis of Tsang and Lindsay (2020), where the gaps in their database do not just limit the analysis, it invalidates the analysis. Indeed, Pallister et al. (2019) argue that:

“Most observatories exist to produce evidence-based, scientific information and forecasts with associated uncertainties in a comprehensible format, i.e., actionable scientific advice ... For rapid and accurate information dissemination, observatories should convey hazard information in standardized formats and use direct modes of communication (e.g., public webpages, e.g., with front page alerts and warnings, social media, and automated messaging). Common formats used by many observatories include color-coded alert or status messages via web pages and in some cases by social media.”

Our findings support this, and highlight the need for the use of such observatory reporting in studies reviewing capabilities in preparing for, responding to and recovering from a volcanic crisis.

Fundamentally, such a gap analysis needs to consider all the information collected and produced by the volcano observatory charged with handling surveillance operations and reporting duties to civil protection, including not just published documents in the scientific literature. Thus, to avoid limits and bias in such a gap analysis observatory-based data sets must be turned to when analysing eruption responses and mitigation efforts. Indeed, the observatories will be only too glad to provide information that can contribute to a valid and useful outcome that is supportive of observatory operations, and which demonstrates the response and mitigation actions implemented during a crisis. To ensure a necessarily comprehensive treatment of the scientific literature has been completed we recommend, in addition to reaching out to observatories to find out what actions they have taken and products they have issued, that a third party expert (a specialist of the site considered) reviews and checks the material used for the gap analysis. 

Finally, as shown here by the length and rigor of our analysis, it is best to focus on a single site and problem, give that site thorough and complete analysis, and complete the analysis in the home language as well as English. Spreading the analysis too thin, and examining too many sites in different cultures and languages, will only reduce the quality of the analysis and the validity of all results.

## Supplementary Information

**Additional file 1.** Correction to the gap analysis of Tsang and Lindsay (2020) for Piton de la Fournaise: List of problems, erroneous statements, incorrectly cited data, and missing data.

## Data Availability

Not applicable.

## Abbreviations

BRGM: Bureau de Recherches Géologiques et Minières
EMZPCOI: Etat Major de Zone et de Protection Civile de l’Océan Indien
GIS: Geographic Information System
GVP: Global Volcanism Program
IPGP: Institut Physique du Globe de Paris
ISSN: International Standard Serial Number
JIR: Journal de l’Ile de la Réunion
ONF: Office National des Forêts
ORSEC: Organisation de la Réponse de Sécurité Civile
OVPF: Observatoire Volcanologique du Piton de la Fournaise
PGHM: Pelotons de Gendarmerie de Haute Montagne
PSS Volcan: Plan de Secours Spécialisé Volcan
CUMP: Cellules d’urgence médico-psychologique
ORA: Observatoire Réunionnais de l’Air
