# Supplementary material for: Reappraisal of gap analysis for effusive crises at Piton de la Fournaise
Source: J Appl Volcanol. 2022 Jan 10;11(1):2. doi: 10.1186/s13617-021-00111-w (PMC8743686; doi:10.1186/s13617-021-00111-w)
Supplement: Supplementary file 1 — Additional file 1. Correction to the gap analysis of Tsang and Lindsay (2020) for Piton de la Fournaise: List of problems, erroneous statements, incorrectly cited data, and missing data. [file 13617_2021_111_MOESM1_ESM.pdf]

Correction to the gap analysis of Tsang and Lindsay (2020) for Piton de la Fournaise: List of problems, erroneous statements, incorrectly cited data, and missing data

As support to our gap analysis, we hereby provide a correction to all erroneous statements made in Tsang and Lindsay (2020). We point to all incorrectly cited and used data, provide the correct citation and data, while also adding further information that aid in providing an appropriate gap analysis for Piton de la Fournaise in terms of preparedness for, response to and recovery from effusive crises.

These corrections apply to the text of Tsang and Lindsay (2020), hereafter T&L, as well as to the two critical Tables of Tsang and Lindsay (2020), i.e., Tables 6 and 12; hereafter termed T&L-6 and T&L-12. These two tables are fundamental and need to be as complete and correct as possible because they respectively give the overview of what is known regarding all effusive events since 1950 at Piton de la Fournaise in terms of impacts, response and recovery (i.e., the data base on which the gap analysis is based), and the gap analysis itself.

Problems with the literature search

Contrary to the analysis of Tsang and Lindsay (2020), we consider all available documentation, including both peer-reviewed papers in English and French, as well as all publically-available OVPF-IPGP reports in the French language. The use of English documentation only results in a serious oversight because, to be of use to local and national civil defence, reporting by the observatory has to be in the country language. Omitting consideration of such observatory-based documentation thus causes a huge gap in the database of Tsang and Lindsay (2020). A complete and rigorous gap analysis is, instead,

given for effusive hazard at Piton de la Fournaise in Table 2 and is very different to the result generated by Tsang and Lindsay (2020) in T&L-12.

Eruptions that do not exist

Three eruptions listed by Tsang and Lindsay (2020) in their Figure 1 and Table 12 did not exist. These are the ones mentioned in 1978, 1980 and December 2004. A full, exhaustive, and accurate, listing of eruptions at Piton de la Fournaise can be found in Peltier et al. (2009), Roult et al. (2012), Michon et al. (2013) and OVPF-IPGP bulletins (ISSN 2610-5101), so we will not repeat these listings here.

Eruptions that did not threaten population and infrastructure

T&L-6 and T&L-12 list two eruptions (May 2004 and "2018") that need to be removed from their gap analysis, as they did not threaten population or infrastructure. In 2018, four eruptions occurred (April; April-June; July and September-November) (see OVPF-IPGP bulletins ISSN 2610-5101, http://www.ipgp.fr/fr/dernieres-actualites/344). It is not clear which of these four events is being referred to in T&L-6 and T&L-12, given that all 2018 eruptions occurred inside the uninhabited Enclos caldera and their lava fronts stopped between 1.8 km and 6.5 km from the road. The July 2018 eruption did, though, cover 400 m of the summit hiking trail so we assume "2018" was included for that reason. We note that the April-May 2018 eruption also required evacuation of observatory monitoring equipment (Harris et al. 2019), but did not impact hiking trails. The May 2004 also did cover a hiking trail. However, if one were to list all the eruptions that impacted the hiking trails since 1950 that would mean including many other events, where among the more recent cases of lava flows cutting hiking trails (not listed in T&L-6) are the events of December 2010, August 2015 and February 2019. Maps of lava flows until 2010 with a focus on the volcano summit showing units that intersected hiking

trails are published in Davoine and Saint-Marc (2016), and can be assessed here (for all flows since 1950) in Figure 1c. As can be seen from Figure 1c, due to the great number of cases that this would entail adding, we do not add these to our Table 1, but instead did not take into consideration the May 2004 and 2018 cases.

Erroneous statements

- Contrary to what Tsang and Lindsay (2020) said, well-established response protocols to mitigate effusive risk have been in place since the first operations of the Piton de la Fournaise volcano observatory (see main text).

- The Observatoire Volcanologique du Piton de la Fournaise has been created in 1979 and not in 1978 as stated wrongly in Tsang and Lindsay (2020).

- The Enclos structure is not a “large valley” as stated in Tsang and Lindsay (2020). It is actually a 13 × 8 km amphitheater-shaped caldera open to the east. Although it is commonly accepted that the Enclos is the most recent large collapse structure visible at Piton de la Fournaise, its mechanisms of formation and respective ages are still debated. It may have been the result of a series of caldera collapses and successive landslides (cf. Bachèlery 1981; Merle and Lénat 2003; Michon and Saint-Ange 2008; Merle et al. 2010), or formed only by major landslides (cf. Duffield et al. 1982; Gillot et al. 1994; Oehler et al. 2004, 2008).

- In T&L-6 it is wrongly stated the 2018 eruption ignited “numerous forest fires” over a period of 7 months. This was not the case. Instead, the September-November 2018 eruption triggered small vegetation (but not forest) fires that were active for a few days.

- In T&L-6 it is wrongly stated the 2007 lava tubes are now a tourist attraction. The tourist attraction statement appears to result from consultation of a caving guide website (see reference 7 in T&L-6). However, access to the tubes in the 2007 lava flow field are officially

forbidden by the *Préfecture* because of the danger posed by their fragility.

- In T&L-6 it is wrongly stated a tourist died on his way to witness the eruption. In regards to fatalities, in 2007 there were two deaths on the volcano. These both were the result of heart attacks, one in May and one in October. However, neither of the 2007 fatalities were due to active lava flow. The fatality of October 2007 was that of a 65-year-old man who died on his way to the top of the volcano. There was no eruption, although the visitor was in the Enclos to which access had been banned following the collapse of the Cratère Dolomieu in April 2007. On May 8, a 68-year-old man died when returning from an overlook site set up for viewing of the 2007 flow field. This happened 8 days after the end of the eruption (Harris and Villeneuve 2018a). A fatality due to interaction with an on-going eruption did occur in August 2003 when a visitor fell into a hot fissure, but this loss is not listed in T&L-6. Risk, death and injuries facing visitors to eruption sites and volcano national parks are another issue entirely. It can cover accidents involving visitors to the hazard, and can occur both during and between eruptive events as well as at and beyond the eruptive site (Blong 1984; Heggie and Heggie 2004; Heggie 2005; Harris 2015). Risk to visitors to the volcano thus requires its own database, analysis and treatment, where recourse to newspaper reports and hospital records is required (cf. Heggie and Heggie 2004; Harris 2015). Incidents of visitor accidents are thus not considered here in our Table 1.

- Evacuation of the population occurred only in April 1977, March 1986, January 2002 and April 2007, and not for all of the Piton la Fournaise lava flows listed in T&L-6 and T&L-12

- The argument that "[…] residents did not return to an evacuation zone during an eruption (e.g. during most Piton de la Fournaise eruptions) […]" given by Tsang and Lindsay (2020) is incorrect for two reasons. First only four eruptions (of 122 eruptions between 1950 and November 2020) led to evacuation of residents at Piton de la Fournaise. Secondly, in 2007,

the inhabitants returned to their houses once it was deemed safe to do so (Morin 2012). Additionally, in 2002 but also in 2007, several inhabitants, through fear of being robbed while their houses were abandoned, refused to leave their homes (Imazpress 2002). This is also in contradiction with the statement of Tsang and Lindsay (2020) “on La Réunion citizens are not averse to evacuating when necessary”. Community reactions are reported in Morin (2012) for the 2002 and 2007 eruptions.

- In T&L-12, the “Lava flow hazard modelling” column for Piton de la Fournaise has “o” for all entries. However, a public report was published in 2012 to evaluate volcanic hazards at Piton de la Fournaise, and in which the OVPF-IPGP lava flow hazard map is given (Di Muro et al. 2012; Davoine and Saint-Marc 2016).

- The supplementary document 1 "Additional file 1 of Lava flow crises in inhabited areas part I: lessons learned and research gaps related to effusive, basaltic eruptions." (https://figshare.com/articles/journal_contribution/Additional_file_1_of_Lava_flow_crises_in_inhabited_areas_part_I_lessons_learned_and_research_gaps_related_to_effusive_basaltic_eruptions/13010888/1) also provides an incorrect statement concerning the analysis of local newspaper reporting during the 2002 eruption and evacuation of the town of Le Tremblet by Harris and Villeneuve (2018a, 2018b). Harris and Villeneuve (2018a, 2018b) do not refer to the 2002 evacuation as stated by Tsang and Lindsay (2020), but focus solely on the 2007 “evacuation” (see main text).

References

Bachèlery P (1981) Le Piton de la Fournaise (Ile de La Réunion). Etude volcanologique, structural et pétrologique. PhD Thesis, Univ. Clermont-Ferrand II

Blong RJ (1984) Volcanic hazards: a sourcebook on the effects of eruptions. Academic Press, Sydney, p 424

Davoine P, Saint-Marc C (2016) A geographical information system for mapping eruption risk at piton de la Fournaise. In: Bachelery P, Lenat J, Di Muro A, Michon L (eds) Active volcanoes of the Southwest Indian Ocean. Springer-Verlag, Berlin, 305-314

Di Muro A, Bachèlery P, Boissier P, Davoine P, Fadda P, Favalli M, Ferrazzini V, Finizola A, Leroi G, Levieux G, Mairine P, Manta F, Michon L, Morandi R, Nave R, Peltier A, Principe C, Ricci T, Roult G, Saint-Marc C, Staudacher T, Villeneuve N (2012) Evaluation de l'aléa volcanique à La Réunion. [online] Available at: http://www.reunion.developpement-durable.gouv.fr/IMG/pdf/Rapport_1erephase_etude_volcan_web_cle534456.pdf

Duffield WA, Stieltjes L, Varet J (1982) Huge landslide blocks in the growth of Piton de la Fournaise, La Reunion, and Kilauea Volcano, Hawaii. Journal of Volcanology and Geothermal Research 12(1-2):147-160

Gillot PY, Lefèvre JC, Nativel PE (1994) Model for the structural evolution of the volcanoes of Réunion Island. Earth Planet. Sci. Lett. 122(3) :291–302. doi:10.1016/0012-821X(94)90003-5

Harris AJL (2015) Basaltic lava flow hazard. In: Papale P, editor. Volcanic hazards, risks and disasters. Amsterdam: Elsevier: pp. 17–46

Harris AJL, Villeneuve N (2018a) Newspaper reporting of the April 2007 eruption of Piton de la Fournaise part 1: useful information or tabloid sensationalism? J Appl. Volcanol. 7:4. https://doi.org/10.1186/s13617-018-0073-1

Harris AJL, Villeneuve N (2018b) Newspaper reporting of the April 2007 eruption of Piton de la Fournaise, part 2: framing the hazard. J Appl. Volcanol. 7:3. https://doi.org/10.1186/s13617-018-0072-2

Harris AJL, Chevrel MO, Coppola D, Ramsey MS, Hrysiewicz A, Thivet S, Villeneuve N, Favalli M, Peltier A, Kowalski P, Di Muro A, Froger JL, Gurioli L (2019) Validation of an integrated satellite-data-driven response to an effusive crisis: the April–May 2018 eruption of Piton de la Fournaise. Annals of Geophysics 61. https://doi.org/10.4401/ag-7972

Heggie TW (2005) Reported fatal and non-fatal incidents involving tourists in Hawaii volcanoes National Park, 1992–2002. Travel Med Infect Dis. 3:123–31

Heggie TW, Heggie TM (2004) Viewing lava safely: an epidemiology of hiker injury and illness in Hawaii volcanoes national park. Wilderness Environ Med. 15:77–81

Imazpress (2002) https://www.ipreunion.com/archives/reportage/0000/00/00/ipreunion,reportage,270.html

Merle O, Lénat JF (2003) Hybrid collapse mechanism at Piton de la Fournaise volcano, Reunion Island, Indian Ocean. J. Geophys. Res. Solid Earth, 108(B3) :1–11.doi:10.1029/2002jb002014

Merle O, Mairine P, Michon L, Bachèlery P, Smietana M (2010) Calderas, landslides and paleo-canyons on Piton de la Fournaise volcano (La Réunion Island, Indian Ocean). J. Volcanol. Geotherm. Res. 189(1) :131–142. doi:10.1016/j.jvolgeores.2009.11.001

Michon L, Saint-Ange F (2008) Morphology of Piton de la Fournaise basaltic shield volcano (La Réunion Island): Characterization and implication in the volcano evolution. J. Geophys. Res. Solid Earth 113(3) :1–19. doi:10.1029/2005JB004118

Michon L, Di Muro A, Villeneuve N, Saint-Marc C, Fadda P, Manta F (2013) Explosive activity of the summit cone of Piton de la Fournaise volcano (La Réunion island): a historical and geological review. Journal of Volcanology and Geothermal Research 264 :117-133

Morin J (2012) Gestion institutionnelle et réponses des populations face aux crises volcaniques : études de cas à La Réunion et en Grande Comore. PhD Thesis. Université de La Réunion

Oehler JF, Labazuy P, Lénat JF (2004) Recurrence of major flank landslides during the last 2-Ma-history of Reunion Island. Bull. Volcanol. 66(7):585–598. doi:10.1007/s00445-004-0341-2

Oehler JF, Lénat JF, Labazuy P (2008) Growth and collapse of the Reunion Island volcanoes. Bull. Volcanol. 70(6):717–742 . doi:10.1007/s00445-007-0163-0

Peltier A, Bachèlery P, Staudacher T (2009) Magma transport and storage at Piton de La Fournaise (La Réunion) between 1972 and 2007: A review of geophysical and geochemical data. Journal of Volcanology and Geothermal Research, 184(1-2):93–108. http://doi.org/10.1016/j.jvolgeores.2008.12.008

Roult G, Peltier A, Taisne B, Staudacher T, Ferrazini V, Di Muro A, the OVPF group (2012) A new comprehensive classification of the Piton de la Fournaise eruptions spanning the 1986-2011 period. Search and analysis of eruption precursors from a broad-band seismological station. J. Volcano. Geotherm., 241-242 :78-104

Tsang SWR, Lindsay JM (2020) Lava flow crises in inhabited areas part I: lessons learned and research gaps related to effusive, basaltic eruptions. J Appl. Volcanol. 9:9. https://doi.org/10.1186/s13617-020-00096-y
